# Pharmacological Evaluation of the Long-Term Effects of Xanomeline on the M_1_ Muscarinic Acetylcholine Receptor

**DOI:** 10.1371/journal.pone.0015722

**Published:** 2010-12-23

**Authors:** Marianne K. O. Grant, Meredith J. Noetzel, Kayla C. De Lorme, Jan Jakubík, Vladimír Doležal, Esam E. El-Fakahany

**Affiliations:** 1 Department of Psychiatry, University of Minnesota Medical School, Minneapolis, Minnesota, United States of America; 2 Department of Pharmacology, University of Minnesota Medical School, Minneapolis, Minnesota, United States of America; 3 Department of Neuroscience, University of Minnesota Medical School, Minneapolis, Minnesota, United States of America; 4 Department of Neurochemistry, Institute of Physiology, Czech Academy of Sciences, Prague, Czech Republic; Biological Research Center of the Hungarian Academy of Sciences, Hungary

## Abstract

Xanomeline is a unique agonist of muscarinic receptors that possesses functional selectivity at the M_1_ and M_4_ receptor subtypes. It also exhibits wash-resistant binding to and activation of the receptor. In the present work we investigated the consequences of this type of binding of xanomeline on the binding characteristics and function of the M_1_ muscarinic receptor. Pretreatment of CHO cells that stably express the M_1_ receptor for 1 hr with increasing concentrations of xanomeline followed by washing and waiting for an additional 23 hr in control culture media transformed xanomeline-induced inhibition of [^3^H]NMS binding from monophasic to biphasic. The high-affinity xanomeline binding site exhibited three orders of magnitude higher affinity than in the case of xanomeline added directly to the binding assay medium containing control cells. These effects were associated with a marked decrease in maximal radioligand binding and attenuation of agonist-induced increase in PI hydrolysis and were qualitatively similar to those caused by continuous incubation of cells with xanomeline for 24 hr. Attenuation of agonist-induced PI hydrolysis by persistently-bound xanomeline developed with a time course that parallels the return of receptor activation by prebound xanomeline towards basal levels. Additional data indicated that blockade of the receptor orthosteric site or the use of a non-functional receptor mutant reversed the long-term effects of xanomeline, but not its persistent binding at an allosteric site. Furthermore, the long-term effects of xanomeline on the receptor are mainly due to receptor down-regulation rather than internalization.

## Introduction

There are five subtypes of muscarinic acetylcholine receptors that vary in their distribution and function. The M_1_ receptor mediates its response to acetylcholine and pharmacological agonists via coupling to the G_q_/G_11_ class of heterotrimeric guanine nucleotide-binding proteins (G proteins). The resultant activation of phospholipase C leads to a subsequent increase in phosphoinositide hydrolysis, which plays a role in cell growth, survival, and differentiation [1], [2]. Of the five cloned subtypes of the muscarinic receptor, the M_1_ subtype is vital for processes involved in learning and memory. Memory deficits such as those seen in Alzheimer’s disease are currently treated with acetylcholinesterase inhibitors. However, this pharmacological approach is endowed with serious untoward effects due to activation of all subtypes of muscarinic receptors by the elevated levels of acetylcholine. A proposed alternative is to administer M_1_-selective muscarinic receptor agonists. Development of such agonists, however, has been hampered by the highly conserved nature of the orthosteric binding domain among the five receptor subtypes [1], [3], [4].

Xanomeline (3-[3-hexyloxy-1,2,5-thiadiazo-4-yl]-1,2,5,6-tetrahydro-1-methylpyridine) is a novel agonist that has been studied extensively due to its high potency and functional selectivity at M_1_ and M_4_ receptors [5]–[7] and its potential in the treatment of cognitive deficits in schizophrenia [8]. In 1997, our laboratory discovered that xanomeline activates the M_1_ muscarinic acetylcholine receptor in a unique wash-resistant manner, unlike other classical muscarinic agonists such as carbachol [9]. Since then, it has been shown that this type of activation is associated with wash-resistant binding [10], [11] and allosteric modulation [12] of the M_1_ receptor. There is evidence that xanomeline interacts reversibly with the orthosteric site, while it binds persistently to the receptor at a different secondary binding domain [10]–[12].

While the unique short-term effects of xanomeline have been studied extensively, the long-term consequences of its persistent binding remain relatively unknown. There is preliminary evidence that although xanomeline wash-resistant receptor activation is reversed over time, its effects in inhibiting binding of radioligands to the orthosteric domain on the receptor were actually *potentiated* [12], [13]. However, the mechanisms underlying these changes have yet to be explored. It is known that prolonged activation of G protein-coupled receptors by conventional reversible agonists can lead to modulation of receptor expression and response to agonists [14], [15]. The unique ability of xanomeline to persistently bind to and activate the M_1_ receptor may similarly result in down-regulation/desensitization of the receptor. Alternatively, persistently-bound xanomeline may induce modification of the receptor conformation over time. We undertook the current study to further evaluate the possible mechanisms involved in the long-term changes observed in receptor binding and function by the wash-resistant component of xanomeline binding to the M_1_ receptor.

## Results

### Concentration-dependent changes in [^3^H]NMS binding to the M_1_ muscarinic receptor by xanomeline pretreatment

**Figure 1:**
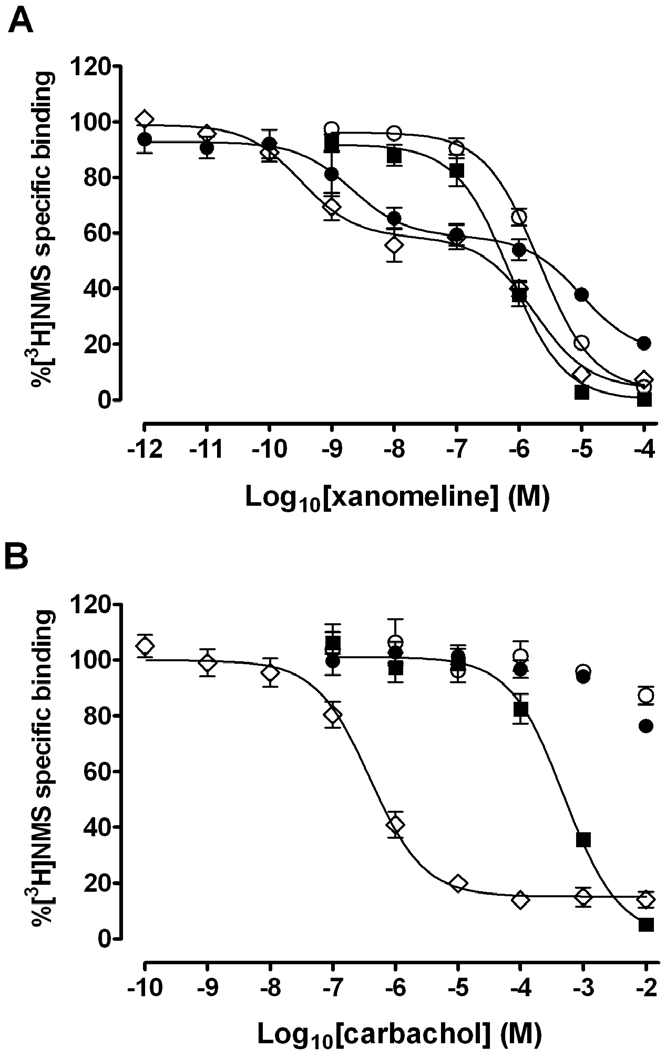
Inhibition of [^3^H]NMS binding by (A) xanomeline or (B) carbachol in CHO cells stably expressing human M_1_ muscarinic acetylcholine receptors. The specific binding of 0.2 nM [^3^H]NMS was measured in the presence of increasing concentrations of agonist in naïve cells (closed squares), or after pretreating with increasing concentrations of agonist for 1 h (open circles) or 24 h (open diamonds) followed by washing and immediate use in the binding assay, or after pretreating with increasing concentrations of agonist for 1 h followed by washing and incubation in agonist-free media for an additional 23 h before use in the binding assay (closed circles). Nonspecific binding was defined by 10 µM atropine. Values represent the means ± standard error of four to six experiments conducted in triplicate.

The specific binding of 0.2 nM [^3^H]NMS to the hM_1_ muscarinic receptor expressed in intact CHO cells was measured in the continuous presence of xanomeline in naïve cells, or following various xanomeline pretreatment and washing conditions. As indicated in Fig. 1A, xanomeline is a potent inhibitor of [^3^H]NMS binding. In accordance with previous reports [12], [17], preincubation of cells with increasing concentrations of xanomeline for 1 h followed by washing away free drug resulted in residual concentration-dependent inhibition of [^3^H]NMS binding, albeit with a lower potency as compared to that observed in naïve cells with xanomeline present in the binding assay mixture. In both cases, the data were best described by a one-site binding model (Table 1).

**Table 1:** Effects of xanomeline or carbachol pretreatments on 0.2 nM [^3^H]NMS binding in CHO hM_1_, rM_1_, or mutant^123^ cells.

	Xanomeline				Carbachol			
	pIC_50_ a	pIC_50_ high b	pIC_50_ low c	I*_max_* d	pIC_50_	I*_max_*		
**CHO hM_1_**								
Control e,f	6.2±0.07			100	3.3±0.01	99±0.3		
1 h washout	5.7±0.05*			97±0.8				
1 h washout/23 h wait		8.6±0.21†(43±2.4%)	5.0±0.06†	84±2.8‡				
24 h washout		9.5±0.13†(43±5.6%)	5.7±0.06	96±0.8	6.3±0.08	85±1.6‡		
**CHO rM_1_**					n.m.^g^			
Control	7.3±0.14			99±0.6				
1 h washout	6.0±0.06*			95±0.5				
1 h washout/23 h wait		7.1±0.16†(74±1.1%)	4.6±0.23†	99±2.3				
24 h washout		9.4±0.20†(47±7.7%)	6.6±0.12	97±0.5				
**CHO rM_1_ mutant^123^**					n.m. g			
Control	7.6±0.14			99±0.5				
1 h washout	6.0±0.02*			96±0.7				
1 h washout/23 h wait	4.9±0.23†			60±7.4‡				
24 h washout	5.8±0.08			90±2.9				

Cells were pretreated with increasing concentrations of xanomeline or carbachol for 1 h or 24 h at 37°C followed by washing and immediate use in the binding assay or further incubation in the absence of free xanomeline for 23 h. Cells were then incubated with 0.2 nM [^3^H]NMS at 37°C for 1 h. Data were corrected for protein as indicated in results. Parameters derived from nonlinear regression analysis are shown as mean ± S.E.M. of four to six experiments conducted in triplicate.

^*a*^Negative logarithm of the IC_50_ for binding to a single affinity site.

^*b*^Negative logarithm of the IC_50_ for the high-affinity agonist binding site; percentage of binding sites shown in parentheses.

^*c*^Negative logarithm of the IC_50_ for the low-affinity agonist binding site.

^*d*^Maximal percentage inhibition of [^3^H]NMS binding.

^*e*^Control, naïve cells were incubated simultaneously with agonist and radioligand.

^*f*^Results shown for xanomeline in control CHO hM_1_ cells are from nonlinear regression analysis with the bottom constrained to be greater than 0.

^*g*^Not measured.

*Significant difference (*p*<0.05) in pIC_50_ between control and 1 h washout as determined by students unpaired *t*-test.

† Significant difference (*p*<0.05) in pIC_50_ between the indicated groups and 1 h washout as determined by one-way ANOVA with Dunnett’s post-test.

‡ Significant difference (*p*<0.05) of I_max_ from 100 percent.

In order to assess the long-term effects of residual xanomeline binding, xanomeline-treated and washed cells were incubated for an additional 23 h in control culture medium. Visually apparent changes in cell density were observed following this pretreatment protocol with xanomeline. In order to account for these variances, the method of Bradford [18] was used to quantify changes in protein content in CHO hM_1_ cells. No changes were evident following short-term exposure to xanomeline. However, concentration-dependent decreases in protein content were observed following 24-h pretreatment with concentrations of xanomeline higher than 100 nM (65±7% maximal decrease at 10 µM xanomeline), or 1-h pretreatment followed by washing and prolonged waiting (32±2% maximal decrease at 10 µM xanomeline). Therefore, the binding data for these experimental groups were adjusted to account for the contribution of cell protein reduction to the observed decrease in [^3^H]NMS binding and are presented in Fig. 1A. Further control experiments were designed to determine if the changes in protein content following long-term xanomeline pretreatments were due to exposure to the xanomeline solvent, dimethylsulfoxide. CHO hM_1_ cells exposed to dimethylsulfoxide in a manner similar to that employed for the various xanomeline treatment protocols did not exhibit significant changes in either protein content or [^3^H]NMS binding (data not shown). Preincubation of cells with increasing concentrations of xanomeline for 1 h followed by washing and waiting for 23 h resulted in the detection of two distinct binding states as determined by nonlinear regression analysis (Fig. 1A and Table 1). Xanomeline IC_50_ at the high-potency binding site that represented approximately half of the total receptor population was three orders of magnitude lower than that observed prior to prolonged waiting. In contrast, the IC_50_ of the lower-potency binding component of xanomeline was comparable to, albeit significantly lower (*p*<0.05) than that at the single site detected without waiting (Table 1). It is also worth noting that maximal inhibition of [^3^H]NMS binding was incomplete at 84±3%. This is in contrast to the complete inhibition of binding observed prior to prolonged waiting. A similar two-site binding profile was evident when cells were preincubated with xanomeline for 24 h followed by washing away free drug, with slightly higher potencies at both sites as compared to 1-h pretreatment followed by washing and waiting 23 h (Fig. 1A and Table 1).

### Comparison of the effects of treatment with carbachol or xanomeline on [^3^H]NMS binding to the M_1_ muscarinic receptor

To test the uniqueness of the observed long-term effects of xanomeline on [^3^H]NMS binding, we repeated the above experiments in CHO hM_1_ cells utilizing the classical reversible muscarinic agonist carbachol. Concentration-dependent decreases in protein content were only observed following 24-h pretreatment with concentrations of carbachol higher than 0.1 mM (26±6.6% maximal decrease at 10 mM carbachol). Therefore, raw data were normalized for protein content. As shown in Fig. 1B, concomitant presence of carbachol and [^3^H]NMS in the binding assay resulted in concentration-dependent and complete inhibition of radioligand binding. Nonlinear regression analysis revealed that the data were best described by a one-site binding model, with a calculated carbachol IC_50_ of ∼500 µM (Table 1). Unlike xanomeline, carbachol pretreatment for 1 h followed by washing away free drug did not result in a significant reduction in [^3^H]NMS binding. Similarly, 1-h preincubation with carbachol followed by washing and 23-h wait caused only a slight decrease in [^3^H]NMS binding at the highest concentration (10 mM) (Fig. 1B). However, 24-h carbachol pretreatment followed by washing away free drug resulted in a concentration-dependent decrease in radioligand binding with a potency three orders of magnitude higher than that observed by exposure of naïve cells to carbachol only during the binding assay (Fig. 1B, Table 1). Nonlinear regression analysis of the data yielded a one-site binding model. This is in sharp contrast to the distinct two binding sites observed following similar pretreatment with xanomeline (Fig. 1A).

### Effects of xanomeline pretreatment on saturation binding of [^3^H]NMS

**Figure 2:**
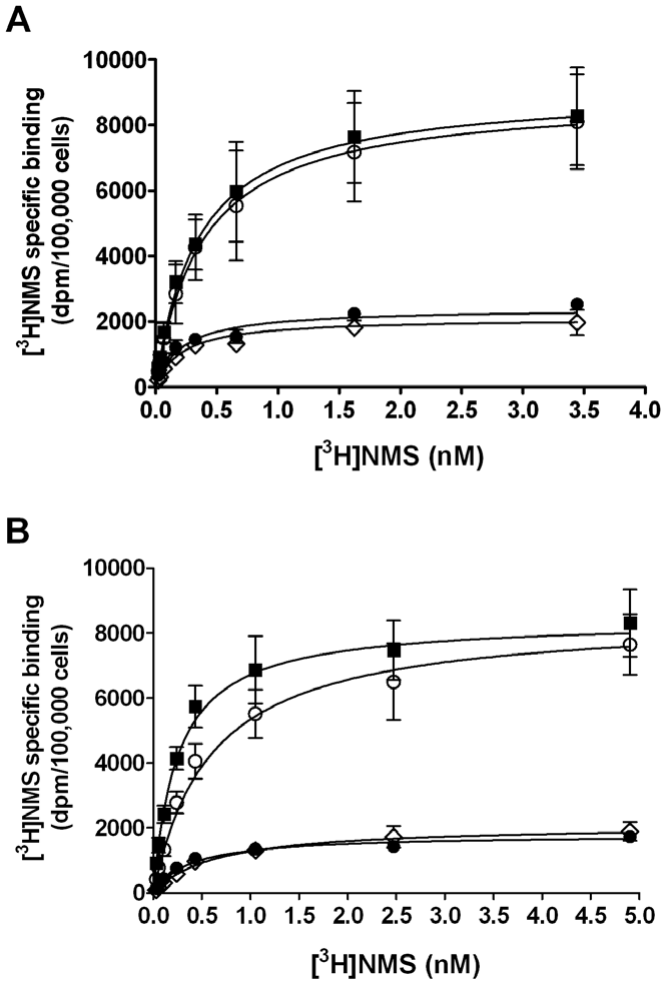
Effects of xanomeline pretreatment, followed by washout, on [^3^H]NMS saturation binding in CHO cells stably expressing human M_1_ muscarinic acetylcholine receptors. Cells were pretreated with (**A**) 300 nM or (**B**) 3 µM xanomeline for 1 h (open circles) or 24 h (open diamonds) followed by washing and immediate use in the binding assay, or after pretreating with xanomeline for 1 h followed by washing and incubation in agonist-free media for an additional 23 h before use in the binding assay (closed circles). Untreated (closed squares) and pretreated cells were subsequently incubated for 1 hour at 37°C with increasing concentrations of [^3^H]NMS. Nonspecific binding was defined by 10 µM atropine. Values represent the means ± standard error of three to ten experiments conducted in triplicate.

Saturation binding experiments were designed to test whether the long-term effects of xanomeline treatments on radioligand binding are the result of reduction in radioligand affinity, maximal binding, or both. The ability of increasing concentrations of [^3^H]NMS to bind to the hM_1_ receptor in untreated CHO hM_1_ cells was compared with that in cells subjected to the various xanomeline pretreatment conditions used in the experiments described above. Pretreatment with 300 nM xanomeline for 1 h followed by washing away free drug did not result in changes in radioligand affinity or maximal cell-surface receptor density (Fig. 2A, Table 2). However, a profound decrease in the maximal binding of [^3^H]NMS was observed 23 h after washing away free drug. The magnitude of this decrease was similar to that detected in cells incubated with the same concentration of xanomeline for 24 h before washing away free drug. Interestingly, a concomitant marked increase in [^3^H]NMS affinity was observed in the latter two groups. Results are summarized in Table 2.

**Table 2:** Effects of xanomeline pretreatment on [^3^H]NMS saturation binding parameters in CHO hM_1_, rM_1_, and mutant^123^ cells.

	300 nM xanomeline		3 µM xanomeline	
	K_D_ a (nM)	B_max_ b	K_D_ (nM)	B_max_
**CHO hM_1_**				
Control^c^	0.35±0.02	9100±1600	0.25±0.03	8500±1000
1 h washout	0.38±0.04	8900±1400	0.59±0.12*	8500±1100
1 h washout/23 h wait	0.21±0.04*	2400±170*	0.38±0.09	1800±150*
24 h washout	0.21±0.04*	2100±250*	0.63±0.17*	2100±380*
**CHO rM_1_**	n.m.^d^			
Control			0.27±0.08	2200±90
1 h washout			0.40±0.04	2600±430
1 h washout/23 h wait			0.30±0.04	1100±150*
24 h washout			0.60±0.12*	1300±150
**CHO mutant^123^**	n.m. d			
Control			0.19±0.02	2700±300
1 h washout			0.42±0.10	3000±380
1 h washout/23 h wait			0.26±0.07	3400±610
24 h washout			0.49±0.11	3600±320

Cells were pretreated with 300 nM or 3 µM xanomeline for 1 h or 24 h at 37°C followed by washing and immediate use in the binding assay or for 1 h followed by washing and further incubation in the absence of free xanomeline for 23 h. Cells were then incubated with increasing concentrations of [^3^H]NMS at 37°C for 1 h. Parameters derived from computer-assisted non-linear regression analysis as described in Methods are presented as mean ± S.E.M. of three to ten experiments conducted in triplicate.

^*a*^Equilibrium dissociation constant for [^3^H]NMS binding.

^*b*^Maximal cell-surface receptor density (dpm/100,000 cells).

^*c*^Control, naïve cells were incubated with radioligand.

^*d*^Not measured.

*ANOVA followed by Dunnett’s post-test detected a significant difference (*p*<0.05) in K_D_ or B_max_ between the pretreated groups compared with vehicle control.

A similar pattern of changes in maximal binding was observed when the concentration of xanomeline used for pretreatments was increased to 3 µM (Fig. 2B). However, decreases in [^3^H]NMS affinity were observed following 1-h and 24-h pretreatment conditions with this concentration of xanomeline. The effects of 1-h pretreatment with xanomeline on radioligand affinity were reduced when washed cells were incubated for 23 h in the absence of free xanomeline (Table 2).

### Effects of xanomeline pretreatment on agonist-stimulated production of inositol phosphates

**Figure 3:**
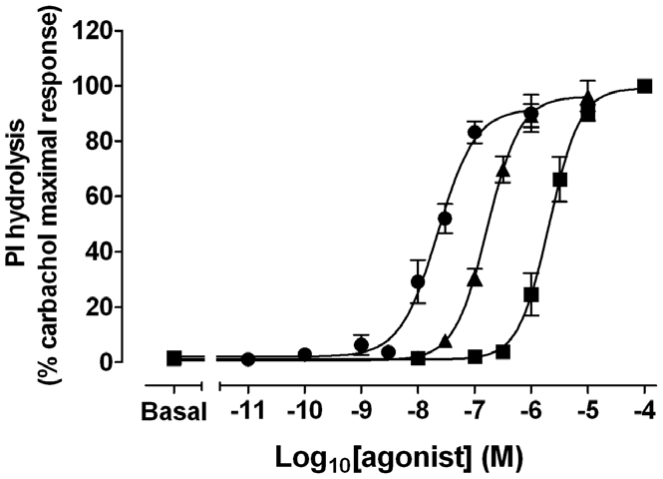
Agonist-mediated PI hydrolysis in CHO cells stably expressing human M_1_ muscarinic acetylcholine receptors. Cells were incubated for 1 h at 37°C with increasing concentrations of carbachol (closed squares), oxotremorine (closed triangles), or xanomeline (closed circles). Results are expressed as percentages of maximal carbachol elicited PI response in untreated cells (11000±800 dpm). Values represent the means ± standard error of three experiments conducted in triplicate.

**Figure 4:**
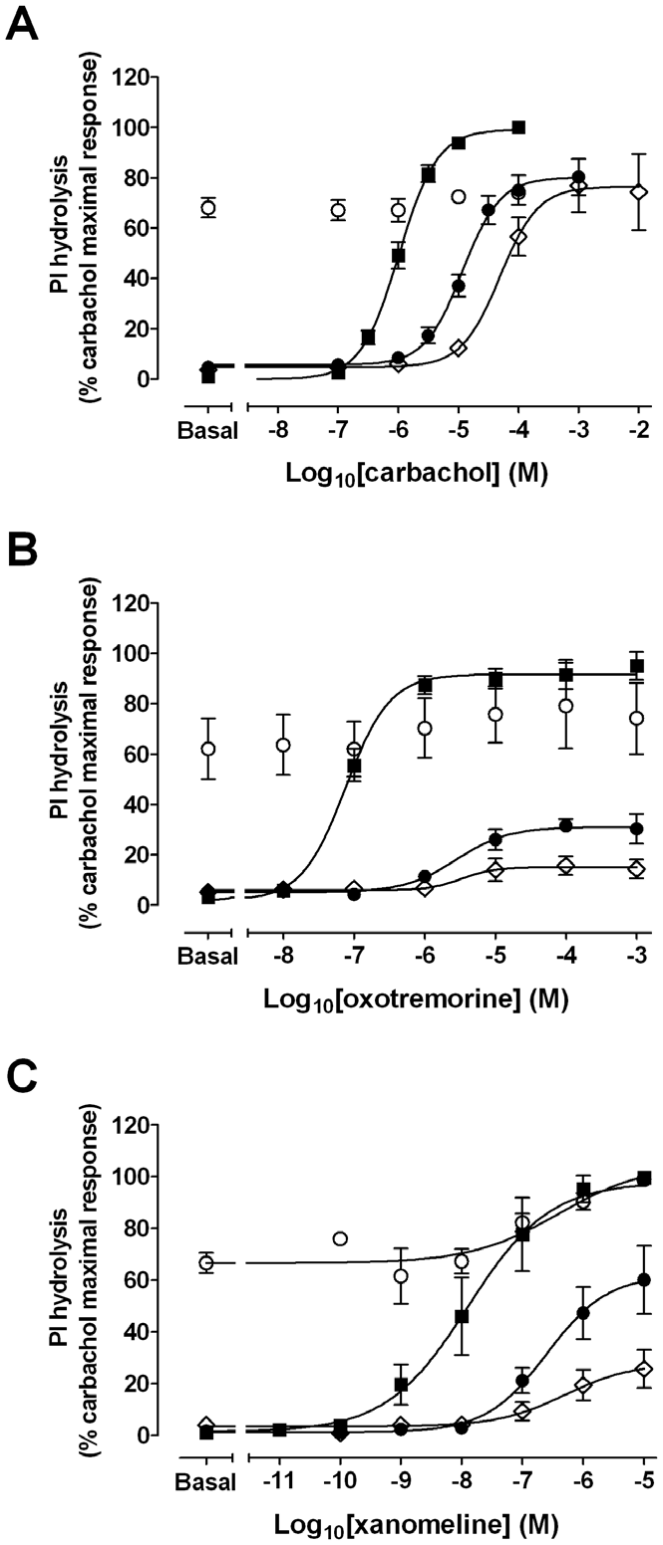
Effects of xanomeline pretreatment, followed by washout, on agonist-stimulated PI hydrolysis in CHO cells stably expressing human M_1_ muscarinic acetylcholine receptors. Cells were pretreated with 300 nM xanomeline for 1 h (open circles) or 24 h (open diamonds) followed by washing and agonist-stimulated PI hydrolysis was measured immediately. Alternatively, cells were pretreated for 1 h followed by washing and incubation in agonist-free media for an additional 23 h (closed circles) before measuring agonist-stimulated accumulation of PI hydrolysis. Sham-treated (closed squares) and xanomeline-treated cells were subsequently incubated for 1 hour at 37°C with increasing concentrations of (A) carbachol, (B) oxotremorine, or (C) xanomeline and accumulation of inositol phosphates was measured. Maximal carbachol induced PI response in untreated cells was (A) 24000±1800 dpm, (B) 8300±900 dpm, (C) 19000±1800 dpm. Values represent the means ± standard error of three to eight experiments conducted in triplicate.

Assays of muscarinic acetylcholine receptor-mediated PI hydrolysis were undertaken to ascertain the functional consequences of wash-resistant xanomeline binding at the hM_1_ muscarinic receptor. Initial experiments were performed to establish concentration-response characteristics of xanomeline in comparison to those of the classical agonists carbachol and oxotremorine in CHO hM_1_ cells (Fig. 3). The maximal accumulation of inositol phosphates induced by xanomeline, carbachol and oxotremorine was similar in magnitude (E*_max_* values of 11000±1100; 11000±800; 10000±800 dpm, respectively). However, xanomeline was more potent than carbachol or oxotremorine (pEC_50_ of 7.6±0.09; 5.7±0.11; 6.7±0.04, respectively). Pretreatment with 300 nM xanomeline for 1 h followed by washing away free drug resulted in a marked increase in basal receptor activity (that corresponded to more than 60% of maximal stimulation by carbachol). Subsequent stimulation of xanomeline-pretreated cells with increasing concentrations of oxotremorine or xanomeline (Figs. 4B and 4C, open circles), but not carbachol (Fig. 4A, open circles) generated further slight increase in PI hydrolysis (in individual experiments). Of particular interest, the effects of xanomeline pretreatment on basal levels were reversed when xanomeline-pretreated cells were incubated in the absence of free xanomeline for 23 h (Fig. 4, closed circles). This reversal was accompanied by a decrease in the maximal responses to carbachol, oxotremorine and xanomeline by 20, 70 and 40%, respectively. The potency of studied agonists to stimulate PI hydrolysis was also reduced as evidenced by 10-, 32- and 11-fold increases in the EC50 for carbachol, oxotremorine and xanomeline, respectively. Similar results were obtained when cells were continuously pretreated with xanomeline for 24 h followed by washing away free drug, although reductions of the maximal responses elicited by xanomeline and oxotremorine were more evident (Fig. 4, open diamonds). Results are summarized in Table 3.

**Table 3:** Effects of xanomeline or carbachol pretreament on activation of PI hydrolysis by carbachol, oxotremorine, or xanomeline in CHO hM_1_ cells.

Pretreatment condition	Agonist stimulation					
	Carbachol		Oxotremorine		Xanomeline	
300 nM xanomeline	pEC_50_ a	E*_max_^b1^*	pEC_50_	E*_max_^b2^*	pEC_50_	E*_max_^ b3^*
Control^c^	6.0±0.06	99±0.5	7.1±0.08	92±4.9	7.9±0.40	98±2.8
1 h washout	n.a.^d^	75±2.6*	n.a.	78±15.6	6.4±0.55*	105±4.5
1 h washout/23 h wait	5.0±0.06*	80±6.9*	5.6±0.09*	31±4.4*	6.6±0.03*	62±14.0*
24 h washout	4.3±0.11*	79±10.7*	5.3±0.29*	15±3.9*	6.3±0.09*	28±7.5*
**10 µM carbachol**						
Control	5.9±0.11	98±1.2	6.8±0.02	98±6.8	7.7±0.11	95±3.7
24 h washout	4.5±0.08†	71±9.9	5.7±0.02†	15±3.9†	6.6±0.01†	18±5.2†

Cells were pretreated with 300 nM xanomeline or 10 µM carbachol for 1 h or 24 h at 37°C followed by washing and immediate use in the functional assay or for 1 h followed by washing and further incubation in the absence of free xanomeline for 23 h. Pretreated or untreated (control) cells were then incubated with increasing concentrations of carbachol, oxotremorine, or xanomeline at 37°C for 1 h and the accumulation of inositol phosphates was determined. Functional parameters were derived from computer-assisted non-linear regression analysis as described in the Methods, and are presented as mean ± S.E.M. of three to nine individual experiments conducted in triplicate.

^*a*^Negative logarithm of the midpoint (potency) parameter.

^*b*^Maximal response. Values are expressed as % maximal response elicited by carbachol in untreated cells (*^b1^* 24000±1800 dpm; *^b2^* 8300±900 dpm; *^b3^*19000±1800 dpm).

^*c*^Control, naïve cells were incubated with agonist.

^*d*^Not applicable.

*ANOVA followed by Dunnett’s post-test detected a significant difference (*p*<0.05) in pEC_50_ or E_max_ between the pretreated groups compared with control.

† Student’s *t*-test detected a significant difference (*p*<0.05) in pEC_50_ or E_max_ between the pretreated groups compared with control.

### Concentration dependence of xanomeline-induced long-term changes in receptor sensitivity

**Figure 5:**
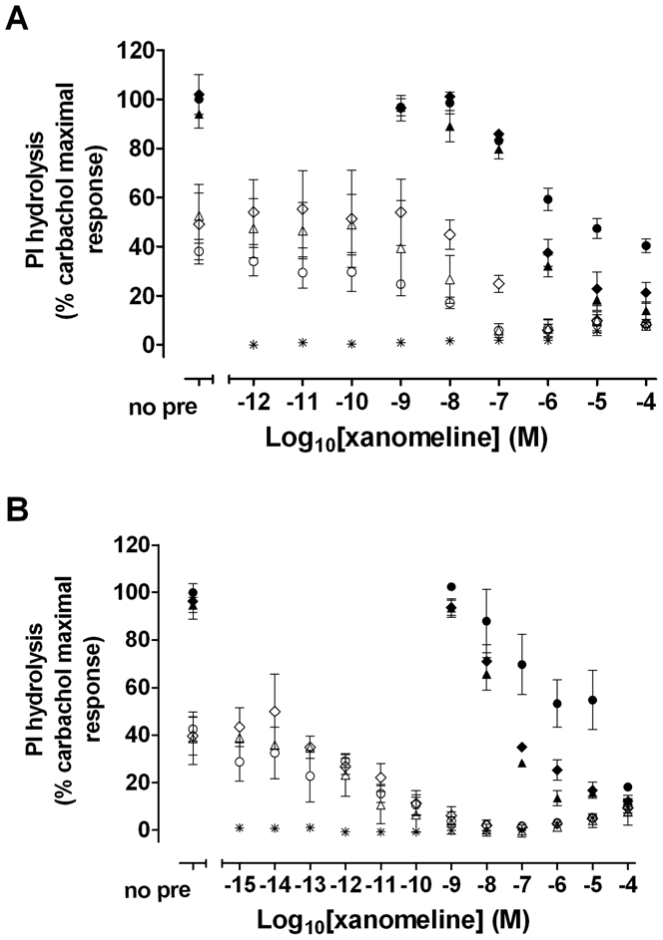
Antagonism of agonist-induced stimulation of PI hydrolysis by xanomeline pretreatment in CHO cells stably expressing human M_1_ muscarinic acetylcholine receptors. Cells were pretreated with increasing concentrations of xanomeline for (A) 1 h followed by washing and incubation in agonist-free media for an additional 23 h, or (B) 24 h followed by washing. Cells were subsequently incubated for 1 h at 37°C in the absence (asterisks) or presence of carbachol at 1 µM (open circles) or 10 mM (closed circles); oxotremorine at 0.1 µM (open triangles) or 1 mM (closed triangles); or xanomeline at 0.03 µM (open diamonds) or 0.1 mM (closed diamonds). Maximal carbachol-induced PI response in untreated cells was (A) 17000±3700 dpm, (B) 20000±1000 dpm. Values represent the means ± standard error of two to four experiments conducted in triplicate.

Additional functional experiments were undertaken to determine the potency of xanomeline in producing its long-term functional effects. CHO hM_1_ cells were subjected to pretreatment with increasing concentrations of xanomeline (1 fM-10 µM) for 1 h followed by washing and waiting for 23 h or pretreatment continuously for 24 h as previously described. Subsequently, cells were stimulated with carbachol, oxotremorine or xanomeline at either EC_50_ (1 µM, 0.1 µM, or 0.03 µM, respectively) or maximal (10 mM, 1 mM, or 0.1 mM, respectively) concentrations. As shown in Fig. 5A (open symbols), pretreatment with xanomeline for 1 h followed by washing and waiting for 23 h resulted in comparable concentration-dependent decreases in PI hydrolysis elicited by EC_50_ concentrations of all three agonists used. However, a more potent concentration-dependent decrease was observed following prolonged continuous pretreatment for 24 h with xanomeline (Fig. 5B, open symbols). The maximal response elicited by all three agonists was also reduced in a concentration-dependent manner following both pretreatment conditions using higher concentrations of xanomeline (µM range) (Figs. 5A and 5B, closed symbols). Interestingly, xanomeline pretreatment for 24 h elicited more potent inhibition of oxotremorine- and xanomeline-mediated PI hydrolysis than the response mediated by carbachol. It is also worth noting that both pretreatment conditions resulted in a slight increase in basal response at xanomeline concentrations of 1 µM and above (Figs. 5A and 5B, asterisks).

### Comparison of the effects of treatment with carbachol or xanomeline on agonist-stimulated production of inositol phosphates

**Figure 6:**
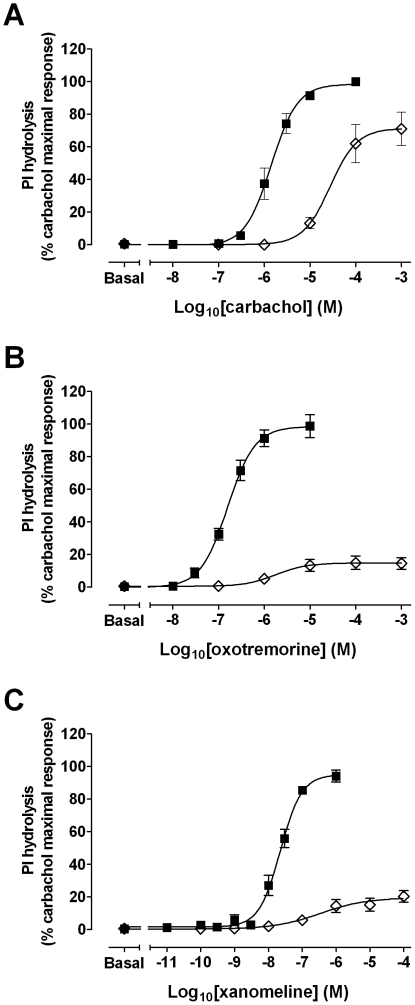
Effects of carbachol pretreatment, followed by washout, on agonist-stimulated PI hydrolysis in CHO cells stably expressing human M_1_ muscarinic acetylcholine receptors. Cells were pretreated without or with 10 µM carbachol for 24 h followed by washing before measuring agonist-stimulated accumulation of inositol phosphates. Untreated (closed squares) and carbachol-treated (open diamonds) cells were subsequently incubated for 1 hour at 37°C with increasing concentrations of (A) carbachol, (B) oxotremorine, or (C) xanomeline and accumulation of inositol phosphates was measured. Results are expressed as percentages of maximal carbachol-elicited PI response in untreated cells (9800±1900 dpm). Values represent the means ± standard error of three experiments conducted in triplicate.

Our radioligand binding data indicate that long-term treatments with xanomeline result in decreases in cell-surface receptor availability (Fig. 2, Table 2). In order to determine if these changes play a role in the long-term functional effects observed following xanomeline pretreatment, additional comparative experiments were conducted following 24-h pretreatment with carbachol. The concentration of carbachol chosen for these experiments was 10 µM, which displays an equi-effective response to that of 300 nM xanomeline (Fig. 3). As can be seen in Figs. 6 and 4, CHO hM_1_ cells pretreated with carbachol for 24 h followed by washing and subsequent agonist stimulation (carbachol, oxotremorine, or xanomeline) exhibited alterations in receptor sensitivity similar to those obtained following 24-h pretreatment with xanomeline. Stimulation of carbachol-pretreated cells with increasing concentrations of oxotremorine or xanomeline resulted in a reduction in maximal response, while the maximal response elicited by carbachol was not changed. Additionally, this treatment resulted in a decrease in the potency of all the agonists used. Results are summarized in Table 3.

### Time dependence of the effects of xanomeline pretreatment on persistent receptor activation and agonist response

**Figure 7:**
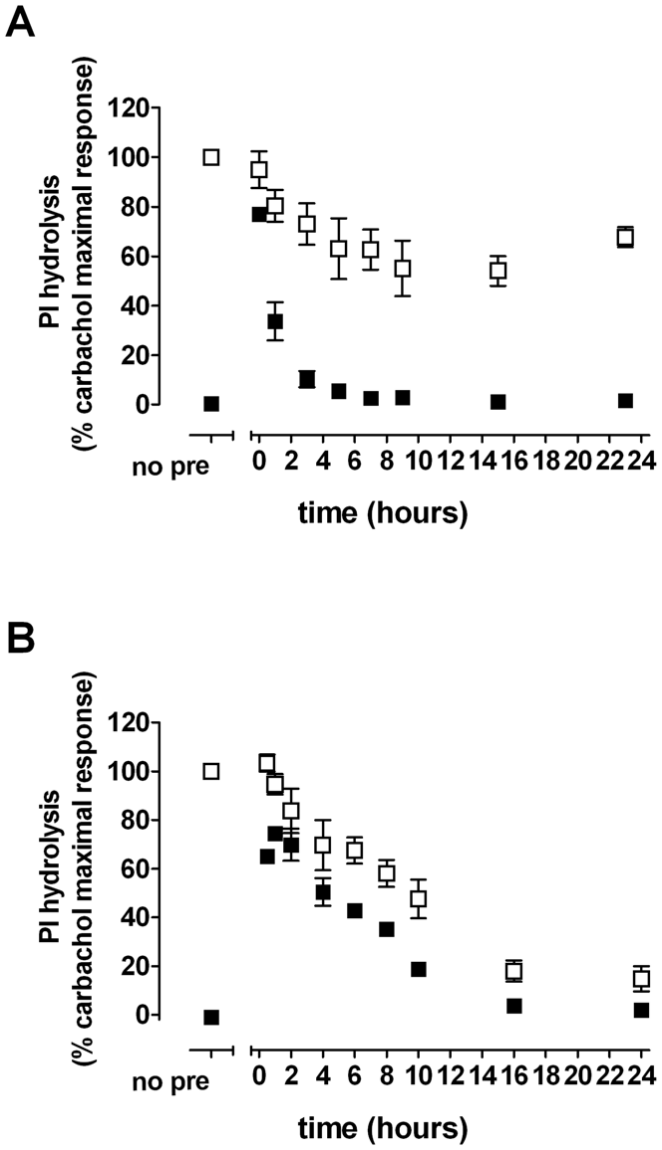
Time dependence of xanomeline-induced persistent activation and antagonism of agonist-stimulated PI hydrolysis in CHO cells stably expressing human M_1_ muscarinic acetylcholine receptors. Cells were pretreated with 300 nM xanomeline for (A) 1 h followed by washing and incubation in agonist-free media for the indicated time periods, or (B) continuously for the indicated time periods before washing. Subsequently, cells were incubated for 1 h at 37°C in the absence of further agonist stimulation (closed squares) or in the presence of 10 µM carbachol (open squares) and accumulation of inositol phosphates was measured. Results are expressed as percentages of maximal carbachol-elicited PI response in untreated cells, which was 32000±2000 dpm and 27000±5000 dpm in case of A and B, respectively. Values represent the means ± standard error of two to three experiments conducted in triplicate.

We have shown that the increase in basal receptor activity observed following pretreatment with xanomeline for 1 h followed by washing is reversed when cells are allowed to incubate for 23 h in the absence of free ligand (Fig. 4). Further experiments were designed to determine the time course of this reversal process. CHO hM_1_ cells were pretreated with 300 nM xanomeline for 1 h, washed, then allowed to incubate for various time periods (0 to 23 h) in ligand-free media. Alternatively, cells were pretreated continuously with xanomeline for various time periods, from 30 minutes to 24 h, prior to washing and immediate use. As shown in Fig. 7A (closed symbols), a significant increase in basal receptor activation of PI hydrolysis was observed when cells were used immediately following 1 h xanomeline pretreatment and washing. However, receptor stimulation elicited by wash-resistant xanomeline binding quickly subsided when cells were allowed to incubate in the absence of free xanomeline, reaching control basal levels within 5 h. While continuous treatment with xanomeline for up to 24 h also resulted in a time-dependent reversal of persistent xanomeline receptor activation, it occurred at a much slower rate (Fig. 7B, closed symbols). In this case, xanomeline-induced stimulation of PI hydrolysis remained elevated for more than 10 h.

We have also shown that pretreatment with xanomeline for 24 h or 1 h followed by washing and waiting for 23 h resulted in an increase in the EC_50_ of carbachol-mediated PI hydrolysis (Fig. 4A). In order to determine the time course of development of this phenomenon, parallel experiments were conducted in each of these two paradigms where CHO hM_1_ cells were subsequently stimulated with 10 µM carbachol. In both experimental designs, the ability of carbachol to elicit PI hydrolysis was markedly reduced over time. As can be seen in Fig. 7A (open symbols), pretreatment with xanomeline for 1 h followed by washing and varied wait periods resulted in a rapid decrease in carbachol-mediated PI hydrolysis, which was maximally reduced by approximately 50% following 9 h of incubation in the absence of xanomeline. However, when cells were continuously exposed to xanomeline for up to 24 h, the ability of carbachol to elicit a response subsided gradually in parallel with that of the observed decline in xanomeline-induced persistent receptor activation, resulting in maximal inhibition of 80% by 24 h of xanomeline exposure (Fig. 7B, open symbols).

### Comparison of the effects of xanomeline pretreatment on [^3^H]QNB and [^3^H]NMS binding

**Figure 8:**
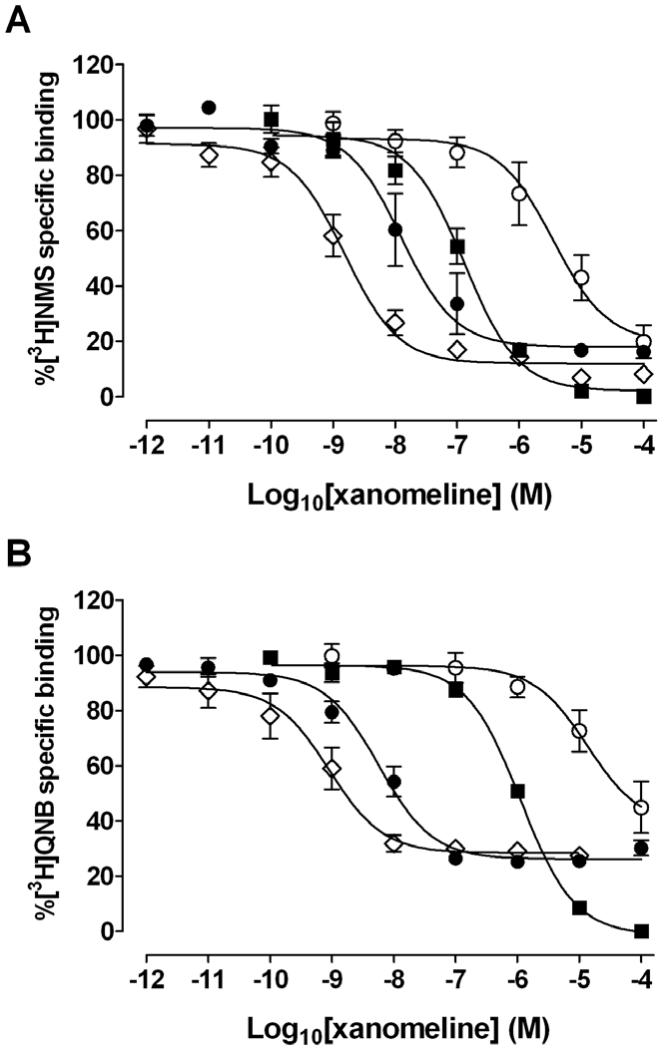
Effects of xanomeline pretreatment, followed by washout, on binding of receptor-saturating concentrations of [^3^H]NMS and [^3^H]QNB in CHO cells stably expressing human M_1_ muscarinic acetylcholine receptors. The binding of (A) 2.9 nM [^3^H]NMS or (B) 1.4 nM [^3^H]QNB was measured in the presence of increasing concentrations of xanomeline in naïve cells (closed squares), or after pretreating with increasing concentrations of xanomeline for 1 h (open circles) or 24 h (open diamonds) followed by washing and immediate use in the binding assay, or after pretreating with increasing concentrations of agonist for 1 h followed by washing and incubation in agonist-free media for an additional 23 h before use in the binding assay (closed circles). Nonspecific binding was defined by 10 µM atropine. Values represent the means ± standard error of three to four experiments conducted in triplicate.

The previously observed decrease in the maximal binding of [^3^H]NMS could be due to either receptor internalization or down-regulation, given that [^3^H]NMS is a permanently-charged quaternary amine that binds only to cell-surface receptors. Therefore, further experiments were designed to compare the long-term effects of xanomeline pretreatment on the specific binding of [^3^H]NMS and [^3^H]QNB, a lipophilic ligand that accesses both cell-surface and internalized (but not degraded) receptors [19]. Receptor-saturating concentrations of both radioligands (2.9 nM [^3^H]NMS; 1.4 nM [^3^H]QNB) were used in order to observe effects on receptor number without interference from changes in radioligand affinity. As shown in Fig. 8, the presence of xanomeline in the binding assay medium with naïve CHO hM_1_ cells resulted in complete inhibition of the binding of both radioligands in a concentration-dependent manner. Pretreatment with xanomeline for 1 h followed by washing away free drug resulted in residual concentration-dependent inhibition of [^3^H]NMS and [^3^H]QNB binding with similar lower potency than that obtained when xanomeline was incorporated in the binding assay medium with naïve cells. Maximal inhibition of binding of either radioligand was incomplete. Incubation of pretreated and washed cells for 23 h in the absence of free xanomeline resulted in marked enhancement of the apparent potency of xanomeline in decreasing binding of both radioligands. These changes in potency were approximately 2.3 and 3.5 orders of magnitude greater than those observed following washing off xanomeline, but prior to prolonged waiting, in the case of [^3^H]NMS and [^3^H]QNB, respectively. Again, maximal inhibition of binding of either radioligand was incomplete. Continuous incubation of cells with xanomeline for 24 h followed by washing away free drug immediately prior to conducting the binding assay resulted in further increase in xanomeline potency in decreasing binding of both radioligands. In all instances, radioligand binding was best described by a one-site model. As previously noted, binding data were adjusted to account for decreases in protein content following long-term pretreatments with xanomeline. Results are summarized in Table 4.

**Table 4:** Effects of various xanomeline treatment conditions on the specific binding of [^3^H]NMS or [^3^H]QNB in CHO hM_1_ cells.

	[^3^H] NMS (2.9 nM)		[^3^H] QNB (1.4 nM)	
	pIC_50_	I*_max_*	pIC_50_	I*_max_*
Control a	6.9±0.08	98±1.0	5.9±0.03	102±1.5
1 h washout	5.5±0.20*	80±3.6*	4.8±0.08*	62±9.6*
1 h washout/23 h wait	7.8±0.38	84±0.4*	8.3±0.19*	74±2.0*
24 h washout	8.8±0.26*	88±0.6*	9.2±0.30*	72±2.2*

Data shown in Fig. 4 were corrected for protein as indicated in results. Parameters derived from nonlinear regression analysis are shown as mean ± S.E.M. of three to four experiments conducted in triplicate. All other details as in Table 1.

*^a^*Control, naïve cells were incubated simultaneously with xanomeline and the radioligands.

*Significant difference (*p*<0.05) in pIC_50_ between the indicated groups and control as determined by one-way ANOVA followed by Dunnett’s post-test.

### Effects of xanomeline pretreatment on the rate of [^3^H]NMS dissociation

Wash-resistant binding of xanomeline to the M_1_ muscarinic receptor results in allosteric modulation of the receptor primary binding domain [10], [12], [19]. This may be reflected in an altered rate of [^3^H]NMS dissociation when dissociation is maximally effected by receptor-saturating concentrations of atropine. Therefore, experiments were designed to determine if the various xanomeline pretreatments have an effect on the dissociation rate constant (*k*_off_) of [^3^H]NMS and hence, if xanomeline is still bound to the receptor following washing and prolonged incubation in the absence of free xanomeline. CHO hM_1_ cells were subjected to the various pretreatments with xanomeline (10 µM), then incubated with 0.5 nM [^3^H]NMS for one hour. Dissociation of the radioligand was initiated by the addition of 10 µM atropine and the dissociation reaction was allowed to proceed for various time intervals. In all instances, radioligand dissociation was best described by a monoexponential model. Pretreatment with xanomeline for 1 h followed by washing resulted in slowing down of the rate of [^3^H]NMS dissociation by 35%. The dissociation rates of [^3^H]NMS remained the same when pretreated cells were further incubated without free xanomeline for 23 h or pretreated continuously for 24 h.

### Role of receptor activation in effecting the long-term changes in receptor binding induced by xanomeline pretreatment

**Figure 9:**
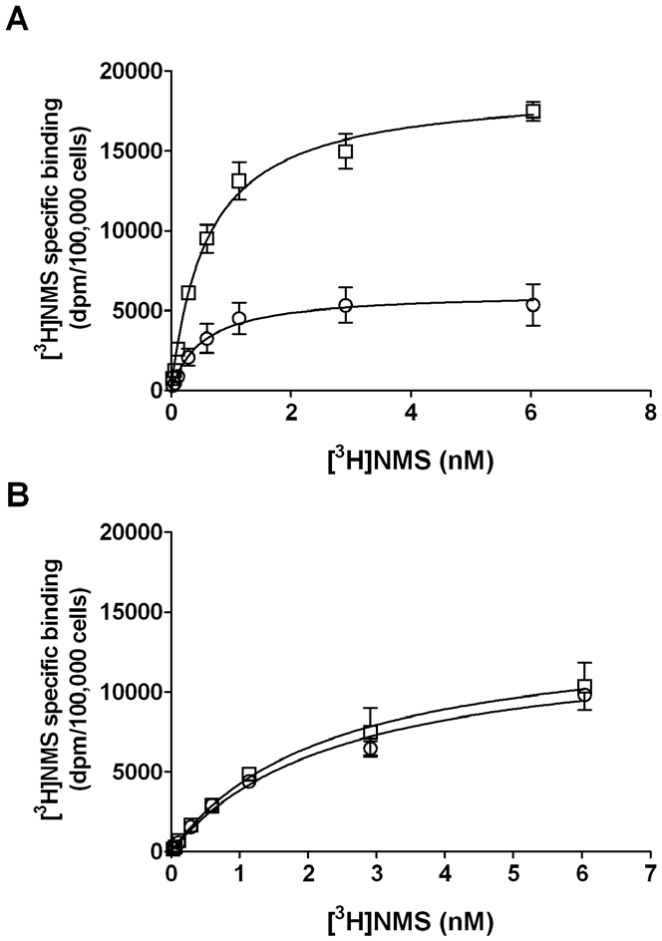
Effects of atropine on the long-term effects of xanomeline pretreatments on [^3^H]NMS saturation binding in CHO cells stably expressing human M_1_ muscarinic acetylcholine receptors. (A) Presence of atropine during 1 h pretreatment followed by 23 h wait in ligand-free media. Cells were pretreated with 3 µM xanomeline in the presence of 10 µM atropine (open circles), or with atropine alone (open squares) for 1 h followed by washing and incubation for 23 h in ligand-free media. (B) Alternatively, atropine was added during the 23 h wait following pretreatment with xanomeline and washing. Cells were pretreated with 3 µM xanomeline (open circles) or sham treated (open squares) for 1 h followed by washing and incubation for 23 h in the presence of 10 µM atropine. For all experiments, cells were subsequently incubated for 1 h at 37°C with increasing concentrations of [^3^H]NMS. Nonspecific binding was defined by 10 µM atropine. Values represent the means ± standard error of four experiments conducted in triplicate.

We have previously shown that while xanomeline wash-resistant binding to the M_1_ receptor takes place at an allosteric domain on the receptor, receptor activation by this mode of xanomeline binding is sensitive to blockade by atropine and therefore involves the receptor orthosteric site [10]–[12]. Therefore, additional binding experiments were designed to determine whether receptor activation is required for the induction of the observed long-term effects of xanomeline in CHO hM_1_ cells. To this end, a receptor-saturating concentration of the muscarinic antagonist atropine (10 µM) was added either simultaneously with xanomeline (3 µM) during the 1-h pretreatment period, or during the 23-h period following washing off free xanomeline. Appropriate atropine controls in the absence of xanomeline were included. Subsequently, [^3^H]NMS saturation binding isotherms were established. As shown in Fig. 9A and Table 5, long-term changes in receptor density were still evident following blockade of the orthosteric site with atropine only during the initial pretreatment period. Furthermore, atropine did not prevent persistent binding of xanomeline to the receptor, supporting the notion that this mode of binding occurs at a secondary site on the receptor [12], [13]. In contrast, when atropine was present only during the 23-h incubation after xanomeline pretreatment and washing, the long-term effects of xanomeline were completely obliterated (Fig. 9B, Table 5).

**Table 5:** Effects of atropine during pretreatment with 3 µM xanomeline or following washout on [^3^H]NMS saturation binding parameters and activation of PI hydrolysis by carbachol in CHO hM_1_ cells.

	[^3^H]NMS binding parameters		PI hydrolysis	
	K_D_ (nM)	B_max_	pEC_50_	E*_max_* a
**Without atropine**				
Control (no pretreatments) b	0.31±0.02	12000±1200	6.07±0.09	101±1.4
1 h xanomeline/washout/23 h wait	0.36±0.02	2400±260*	5.36±0.12*	98±8.9
**Presence of atropine during 1 h pretreatment**				
1 h sham + atropine/washout/23 h wait	0.52±0.09	19000±800	5.87±0.09	100±18
1 h xanomeline + atropine/washout/23 h wait	0.55±0.02	6100±1300*	5.87±0.10	100±17
**Presence of atropine during 23 h wait**				
1 h sham/washout/23 h wait + atropine	2.4±0.31	14000±2700	4.42±0.07	89±7.8
1 h xanomeline/washout/23 h wait + atropine	2.6±0.49	13000±800	4.32±0.04	110±14

Cells were pretreated for 1 h with xanomeline and/or atropine followed by extensive washing and waiting for 23 h in the absence or presence of atropine. Parameters derived from nonlinear regression analysis of data shown in Fig. 9 are presented as mean ± S.E.M. of three experiments performed in triplicate. All other details as for Tables 2 and 3.

*^a^*Expressed as percentage of the maximal response to carbachol in untreated cells (25000±2200 dpm).

*^b^*Control, naïve cells were incubated with radioligand in binding assays, or carbachol in functional assays.

*ANOVA followed by Tukey’s post-test detected a significant difference (*p*<0.05) between the xanomeline pretreated groups compared with respective control/sham treatment.

We also used specific receptor mutants to further prove a role of receptor activation in xanomeline-mediated receptor regulation. Our laboratory has previously shown that point mutation of arginine-123 in the sequence of the rat M_1_ receptor results in nearly complete loss of receptor responsiveness to agonists without significant changes in receptor binding properties [21]. We utilized this receptor mutant (mutant^123^) expressed in CHO cells to determine if a functional receptor is necessary to elicit the long-term effects of xanomeline on radioligand binding. However, because this mutation was done in the rat M_1_ receptor sequence, necessary control experiments were performed in CHO cells expressing rat wild-type M_1_ receptor (rM_1_) for comparison. Xanomeline displayed a higher potency than carbachol in stimulating PI hydrolysis in CHO rM_1_ cells (pEC_50_ = 7.4±0.11 and 5.6±0.10, respectively), with a slightly lower maximal response (E*_max_* = 18000±5500 dpm for xanomeline; 23000±6100 dpm for carbachol). In agreement with previous findings [21], the mutant^123^ receptor did not produce a significant PI response following stimulation with carbachol or xanomeline (data not shown).

As an additional control, CHO rM_1_ or mutant^123^ cells were treated with 1 mM carbachol for 24 h to induce receptor down-regulation [15], [22]. Subsequent ability of 0.2 nM [^3^H]NMS to bind to the receptor was compared. As expected, [^3^H]NMS binding in rM_1_ cells was reduced by approximately 95% (data not shown). In contrast, no significant reduction in [^3^H]NMS binding was observed in mutant^123^ cells, supporting the notion that agonist-induced receptor regulation is indeed contingent on receptor activation (data not shown).

**Figure 10:**
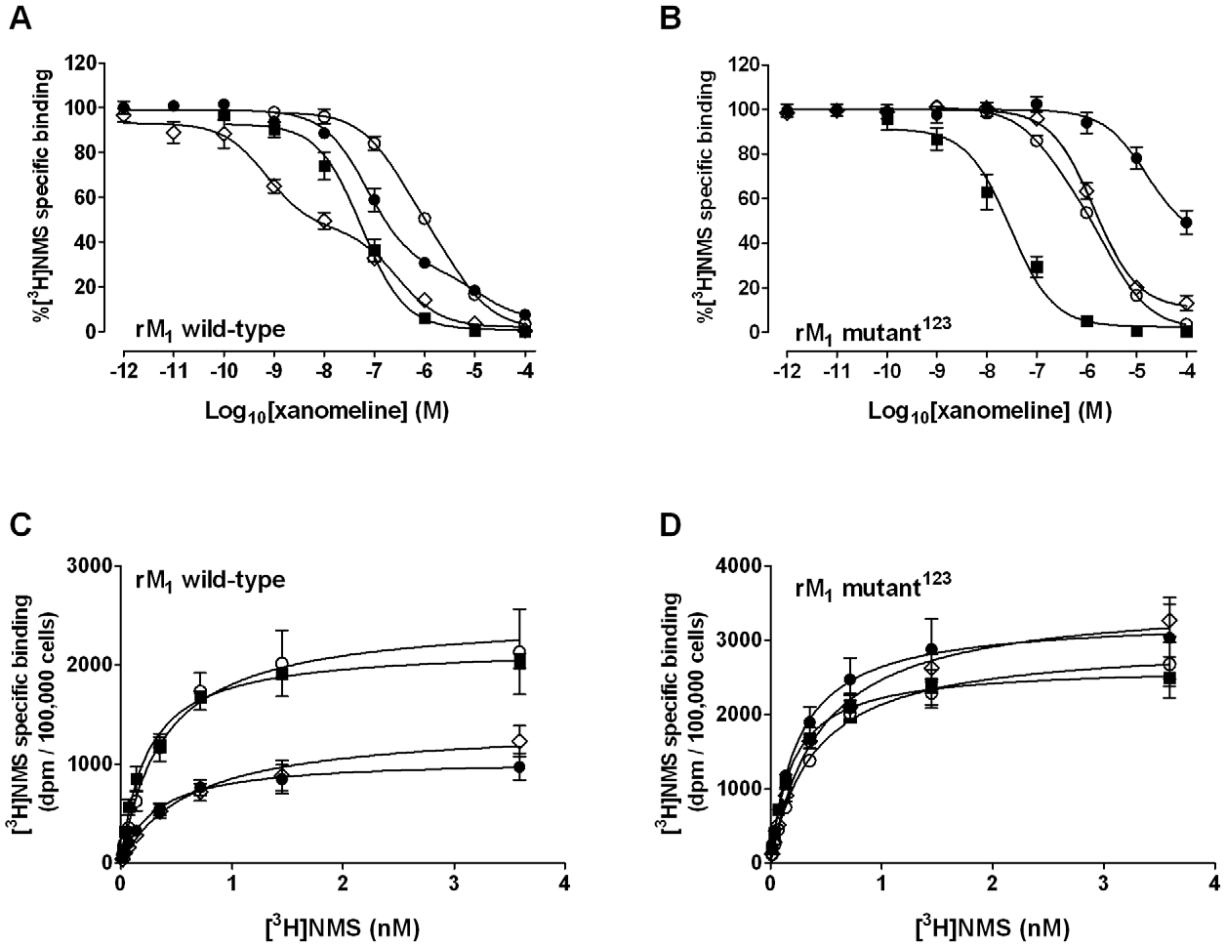
Effects of xanomeline pretreatment, followed by washout, on [^3^H]NMS binding in CHO cells stably expressing rat M_1_ wild-type or rat M_1_ mutant^123^ muscarinic acetylcholine receptors. For all figures, radioligand binding assays were performed for 1 hour at 37°C using naïve cells (closed squares), or after pretreating with xanomeline for 1 h (open circles) or 24 h (open diamonds) followed by washing and immediate use in the binding assay, or after pretreating for 1 h followed by washing and incubation in agonist-free media for an additional 23 h before use in the binding assay (closed circles). Top row: Inhibition of binding of 0.2 nM [^3^H]NMS, was measured in (A) rat wild-type or (B) rat mutant^123^ cells in the presence of increasing concentrations of xanomeline in naïve cells or after pretreating with increasing concentrations of xanomeline. Bottom row: Saturation binding of [^3^H]NMS in (C) rat wild-type or (D) rat mutant^123^ cells. Cells were pretreated with 300 nM xanomeline as described above and were subsequently incubated with increasing concentrations of [^3^H]NMS. Nonspecific binding was defined by 10 µM atropine. Values represent the means ± standard error of four to seven experiments conducted in triplicate.

Experiments measuring the decrease in binding of 0.2 nM [^3^H]NMS in mutant^123^ cells following the various xanomeline pretreatment conditions were compared with those in rM_1_ cells. As shown in Figs. 10A and 10B, and Table 1, xanomeline bound with similar high potency (IC_50_ of approximately 50 nM) to both the wild-type rM_1_ and mutant^123^ receptors in naïve cells. Additionally, short-term xanomeline wash-resistant binding in mutant^123^ cells was virtually identical to that observed in rM_1_ cells. However, the long-term effects of xanomeline on [^3^H]NMS binding evident in rM_1_ cells were drastically attenuated by this mutation. In agreement with our findings in CHO hM_1_ cells, nonlinear regression analysis of data from rM_1_ cells resulted in a two-site binding model for both long-term treatments, albeit at lower potencies (Table 1). In contrast, data from the mutant^123^ cells were best described in terms of a simpler one-site model of binding in all cases (Fig. 10B, Table 1). Furthermore, continuous pretreatment with xanomeline for 24 h followed by washing resulted in a binding profile indistinguishable from that following short-term xanomeline pretreatment, although maximal inhibition of [^3^H]NMS binding was incomplete at 92%. Protein content was unaffected by long-term xanomeline pretreatments in rM_1_ and mutant^123^ cells.

Similar findings were obtained in [^3^H]NMS saturation binding experiments. As can be seen in Fig. 10C and 10D, stripping the receptor of function completely annulled the changes in receptor density observed in rM_1_ cells. In contrast, all xanomeline-induced changes in radioligand affinity observed in rM_1_ cells were conserved in mutant^123^ cells (Table 2).

### Role of the orthosteric site in long-term changes in receptor sensitivity induced by xanomeline pretreatment

**Figure 11:**
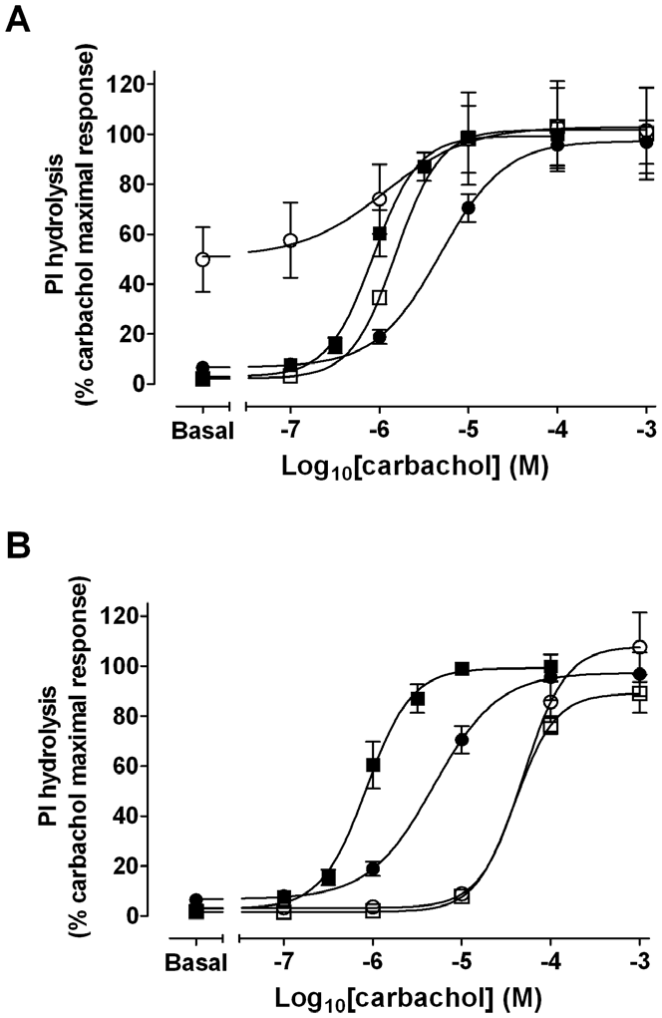
Atropine sensitivity of the long-term effects of xanomeline pretreatments on carbachol-stimulated PI hydrolysis in CHO cells stably expressing human M_1_ muscarinic acetylcholine receptors. (A) Presence of atropine during the initial 1 h pretreatment period. Cells were pretreated with 300 nM xanomeline for 1 h in the absence (closed circles) or presence of 10 µM atropine (open circles) followed by washing and incubation in ligand-free media for 23 h. (B) Effects of atropine presence during the 23 h incubation period following xanomeline pretreatment and washing. Cells were pretreated with 300 nM xanomeline for 1 h followed by washing and incubation for 23 h in the absence (closed circles) or presence of 10 µM atropine (open circles). In both figures, control atropine pretreatments were conducted in the absence of xanomeline pretreatments (open squares). Untreated (closed squares) and treated cells were subsequently incubated with increasing concentrations of carbachol for 1 h at 37°C and accumulation of inositol phosphates was measured. Results are expressed as percentages of maximal carbachol elicited PI response in untreated cells (25000±2200 dpm). Values represent the means ± standard error of three to seven experiments conducted in triplicate.

Experiments measuring PI hydrolysis in CHO hM_1_ cells were designed utilizing the muscarinic antagonist atropine to determine the role of the receptor orthosteric site and receptor activation in the long-term effects of xanomeline on receptor response to agonists. As in binding studies, CHO hM_1_ cells were pretreated with 300 nM xanomeline in the absence or in the presence of 10 µM atropine, either during the 1-h pretreatment period or the 23-h incubation period after free xanomeline had been washed away. Appropriate atropine pretreatment controls in the absence of xanomeline were included. Subsequently, cells were stimulated with increasing concentrations of carbachol. Interestingly, the presence of atropine during the initial 1-h pretreatment with xanomeline preserved xanomeline wash-resistant activation of the receptor (Fig. 11A). However, this pretreatment condition prevented xanomeline-induced changes in carbachol potency or maximal activation of PI hydrolysis. This is contrary to results obtained in binding studies, where blockade of the orthosteric site during the initial pretreatment with xanomeline did not obliterate long-term changes in receptor number (Fig. 9A). In accordance with saturation binding studies (Fig. 9B), the long-term attenuating effects of xanomeline on the response to carbachol were abolished in the presence of atropine during the 23-h incubation period following xanomeline pretreatment and washout (Fig. 11B). Noteworthy, prolonged pretreatment with atropine alone caused a marked shift in EC_50_ of carbachol-induced accumulation of inositol phosphates, in spite of washing off free atropine**.** This change corresponds to the decrease in the affinity of [^3^H]NMS following similar treatment conditions (Fig. 9B, Table 5).

## Discussion

In agreement with previous findings, we have shown that xanomeline binds to and activates the hM_1_ acetylcholine receptor in a wash-resistant manner [9]–[11], [14]. Our current results also indicate that persistent binding of xanomeline to the M_1_ muscarinic receptor elicits additional long-term alterations in radioligand binding to the M_1_ receptor in the absence of free drug. Understanding of these effects is of prime importance in relation to the chronic use of xanomeline in the treatment of schizophrenia [8]. Long-term exposure of cells to xanomeline was accompanied by loss of persistent activation of hydrolysis of inositol phosphates by xanomeline in conjunction with attenuation of receptor activation by other agonists. Possible interpretations of these observations include decreased receptor availability, modifications in receptor conformation, or blockade of the receptor by persistently-bound xanomeline. Any of these effects would result in diminishing radioligand binding in addition to suppressing agonist-mediated activation of the M_1_ receptor.

We have currently shown that acute, as well as chronic, pretreatment with xanomeline results in long-term changes in [^3^H]NMS binding to M_1_ receptors. Previous reports have indicated that similar long-term changes can occur following exposure to xanomeline for as little as 1 minute [14]. Comparisons with carbachol were made in the current study in order to assess whether these effects are unique to xanomeline. As can be seen in Figs. 1A and 1B, xanomeline pretreatments resulted in changes in radioligand binding very distinct from those induced by carbachol. Exposure of cells to xanomeline for 1 h followed by washing resulted in a concentration-dependent decrease in [^3^H]NMS binding with a slightly lower potency than that seen in untreated cells subjected to radioligand binding in the presence of xanomeline. This is in contrast to results obtained using carbachol for pretreatment followed by washing, where no change in radioligand binding was observed. Receptor internalization and down-regulation induced by sustained exposure to conventional reversible agonists are well-documented phenomena [15], [16], [23], [24]. In accordance with these findings, pretreatment with carbachol for 24 h resulted in a marked decrease in [^3^H]NMS binding. The resultant single high-potency binding profile following carbachol long-term treatment was in sharp contrast to the biphasic curve obtained following 24-h xanomeline pretreatment. Interestingly, a similar biphasic curve resulted following pretreatment with xanomeline for 1 h followed by washing and waiting 23 h in xanomeline-free media. Again, this is unlike results obtained using carbachol for pretreatment, where no effect on radioligand binding was observed under these conditions. In fact, previous literature has shown that the marked decrease in binding elicited by 12-h carbachol pretreatment is fully reversed following washing and incubation in carbachol-free media for 24 h [22]. These observations provide further evidence that xanomeline interacts with the M_1_ receptor in a manner unlike other classic muscarinic agonists.

Continuous prolonged incubation of cells with either xanomeline or carbachol reduced receptor sensitivity in responding to activation by agonists. As shown in Fig. 4, pretreatment with 300 nM xanomeline for 24 h resulted in antagonism of the response to carbachol, oxotremorine and xanomeline, as evidenced by a reduction in potency. This was accompanied by a marked decrease in the maximal response of only the latter two agonists. Nearly identical results were obtained when 10 µM carbachol was used for pretreatment (Fig. 6, Table 3). These effects are commensurate with the occurrence of comparable receptor internalization or down-regulation under these pretreatment conditions (data not shown). However, it is interesting to note that pretreatment with either ligand for 24 h results in a greater effect on maximal PI hydrolysis elicited by oxotremorine or xanomeline than on that stimulated by carbachol (Figs. 4, 5B, and 6). While we have currently shown that both oxotremorine and xanomeline *appear* as full agonists in our high receptor expression system (Fig. 3), previous literature has suggested that these ligands may be partial agonists at the M_1_ receptor [25], [26]. This is supported by our observation that xanomeline and oxotremorine exhibit a lower maximal PI response than carbachol in rat wild-type M_1_ cells (data not shown) that express a lower number of receptors compared to human M_1_ cells (Table 2). While the maximal response to the full agonist carbachol should not be affected by a reduction in receptor number in a high receptor expression system due to the presence of spare receptors, the response to partial agonists should be reduced, as full receptor occupancy is necessary for such agents to elicit a maximal response [27].

The biphasic nature of the [^3^H]NMS binding displacement curve following long-term treatments with xanomeline may suggest that low and high concentrations of xanomeline result in differential modes of receptor regulation. At low concentrations of xanomeline (less than 300 nM), down-regulation or internalization may be the predominant mechanism occurring to explain the appearance of a high-potency phase of inhibition of [^3^H]NMS binding following treatment with xanomeline for 24 h or 1-h pretreatment followed by washing and 23-h wait. Pretreatment with increasing concentrations of carbachol for 24 h results in highly potent, monophasic inhibition of 0.2 nM [^3^H]NMS binding (Fig. 1B). Additionally, [^3^H]NMS saturation binding experiments show that maximal receptor density is significantly reduced following both protocols of pretreatment with 300 nM xanomeline. As can be seen in Figs. 2A, 2B and 8, effects of xanomeline on receptor number is saturable. This may account for the inflection of the inhibition of [^3^H]NMS binding in cells pretreated with increasing concentrations of xanomeline for 24 h or for 1 h followed by washing and waiting for 23 h in the absence of free xanomeline. Saturation binding of [^3^H]NMS following 1-h pretreatment with an intermediate concentration of xanomeline (300 nM), washing and waiting for 23 h or treatment for 24 h with this concentration results in an *increase* in [^3^H]NMS affinity (Table 2). This concentration of xanomeline coincides with the end of the long plateau observed in displacement binding experiments. This increase in [^3^H]NMS affinity may mask further decreases in receptor availability occurring at concentrations within this range and contribute to the appearance of the plateau observed in Fig. 1A.

[^3^H]NMS is cell impermeable due to its permanently-charged quaternary amine nature. Thus, the observed decrease in the B_max_ of [^3^H]NMS binding by pretreatment with xanomeline for 24 h before washout, or for 1 h followed by washing and 23-h incubation in agonist-free medium could be due to either receptor internalization or down-regulation. In order to differentiate between these possibilities, further experiments were designed to compare the concentration-dependent effects of xanomeline on the binding of saturating concentrations of [^3^H]NMS and [^3^H]QNB. While [^3^H]NMS labels only cell-surface receptors, [^3^H]QNB is lipophilic and could label both cell-surface and internalized, but not degraded, receptors [19]. As shown in Fig. 8, xanomeline completely inhibits the binding of both [^3^H]NMS and [^3^H]QNB in untreated cells. Similarly, short-term pretreatment with xanomeline followed by washing results in comparable wash-resistant effects on both radioligands, suggesting that acute persistent binding of xanomeline does not result in receptor internalization. The enhanced potency of xanomeline in decreasing binding of either radioligand observed after 24-h pretreatment or 1-h pretreatment followed by washing and waiting for 23 h supports the notion that the long-term effects of xanomeline are likely due to receptor degradation, where the receptors are no longer available to either radioligand. However, the observed similar incomplete inhibition of binding of either radioligand under the latter two conditions suggests that a portion of the cell-surface receptor population is not susceptible to regulation by xanomeline. We cannot exclude the possibility of a time-dependent potentiation of xanomeline-induced negative allosteric effects on the binding of radioligands to cell-surface or internalized (but intact) receptors, particularly due to the greater potentiation of xanomeline effects on [^3^H]QNB than [^3^H]NMS binding. According to the ternary model of receptor allosterism [28], the magnitude of modulation of ligand binding at the receptor orthosteric domain by a given allosteric agent differs from one ligand to another.

In addition to down-regulation/internalization of the receptor, long-term pretreatment with high concentrations of xanomeline (µM range) results in additional modifications of the receptor. Experiments measuring the binding of a low concentration of [^3^H]NMS following long-term pretreatments with xanomeline demonstrated a two-site binding profile with a very distinct plateau separating the two potency states (Fig. 1A). In contrast, similar treatments with xanomeline exhibit a single high-potency component in inhibiting the binding of receptor-saturating concentrations of either [^3^H]QNB or [^3^H]NMS (Fig. 8). The former radioligand binding protocol reflects decreases in either maximal binding or the affinity of the radioligand for the receptor, while the latter condition mainly detects effects on receptor number.

In contrast, long-term incubations with a high concentration of xanomeline (3 µM) leads to a decrease in [^3^H]NMS affinity obtained from saturation binding experiments (Table 2). Previous literature has shown that acute treatment with xanomeline results in allosteric modulation of the M_1_ receptor [9], [11], [19]. We have currently shown that acute as well as chronic pretreatment with xanomeline results in changes in the dissociation rate of [^3^H]NMS, which is one indicator that an allosteric interaction might be occurring. The observed divergent effects of xanomeline pretreatment on the affinity of [^3^H]NMS binding may therefore suggest that xanomeline exerts concentration-dependent allosteric effects.

Previous reports have shown that xanomeline persistently activates the M_1_ receptor following pretreatment with xanomeline for as little as 1 minute followed by the removal of free and reversibly bound agonist [14]. Currently, we have shown that xanomeline-induced persistent activation peaks following 1-h pretreatment, then slowly decreases over time until no functional activity is detected following 16 to 24 h of chronic xanomeline treatment (Fig. 7B). Furthermore, as the duration of xanomeline exposure is lengthened, the ability of carbachol to mediate a response slowly decreases in parallel to the observed reduction in xanomeline persistent receptor activation. Taken together, our data suggest that chronic treatment with xanomeline results in slow desensitization of the PI response, in conjunction with receptor down-regulation. This is similar to previous findings using carbachol to induce agonist-mediated receptor desensitization [22] and xanomeline-induced receptor internalization observed by confocal microscopy [29].

In contrast to results obtained following chronic xanomeline exposure [13], [14], [29], a quick reversal of persistent receptor activation was observed when cells were allowed to incubate in the absence of free xanomeline following 1-h pretreatment, where less than 7 h were necessary to reach control basal activity levels (Fig. 7A). Therefore, the observed differences in receptor desensitization between chronic xanomeline pretreatment and 1- h pretreatment with varied incubation times in ligand-free media may be due to a lower concentration of xanomeline at the receptor biophase under the latter condition. Interestingly, however, virtually identical decreases in receptor availability occur following 24-h incubation with xanomeline or 1-h pretreatment followed by washing and waiting for 23 h. The fact that acute pretreatment with xanomeline followed by washing results in divergent long-term effects on receptor down-regulation and desensitization is further evidence that the wash-resistant component of xanomeline may act to allosterically modulate the M_1_ receptor.

Our data provide evidence that the long-term effects of xanomeline are dependent upon actions at the orthosteric site that lead to functional activation of the receptor. Blockade of the orthosteric site with atropine during the 23-h wait following washing off free xanomeline abolished the long-term changes in B_max_ as well as the functional antagonism of carbachol-mediated PI hydrolysis induced by xanomeline (Figs. 9B and 11B). However, changes in B_max_ were still evident when atropine was only present during the initial pretreatment period (Fig. 9A). Taken together, these data suggest that actions of xanomeline at the orthosteric site during the waiting period (following washing off free xanomeline) are necessary to elicit these long-term changes. It is also important to note that the presence of atropine during the initial pretreatment period does not prevent the persistent binding of xanomeline to the receptor, as long-term changes in B_max_ were still evident. This is in agreement with previous findings that atropine does not interfere with the formation of xanomeline wash-resistant binding [12], [20]. However, in addition to properties as an antagonist, atropine is known to be an inverse agonist [30], [31]. Our current data suggest that prolonged incubation with atropine alone followed by washing results in marked changes in [^3^H]NMS affinity and maximal binding as well as antagonism of carbachol-mediated PI hydrolysis (Figs. 9 and 11). While the co-incubation of atropine with xanomeline during the 23-h wait period resulted in similar effects on binding and function, divergent effects were observed when atropine was present during the initial pretreatment period. Xanomeline-induced changes in binding were unaffected by the presence of atropine during the initial pretreatment period, whereas the functional effects of xanomeline on carbachol potency were abolished. An increase in basal response was also observed in this pretreatment paradigm, further complicating interpretation of these results.

Several other pieces of evidence support the need for a functional receptor to elicit long-term changes observed following long-term treatments with xanomeline. Our laboratory has previously reported that point mutation of arginine-123 in the rat M_1_ receptor sequence results in nearly complete loss of receptor function [21]. Using this cell line, we have shown that a functional receptor is not necessary for xanomeline persistent binding to occur (Fig. 10 B). However, the appearance of a second high-potency binding site is not evident following long-term treatments in the mutant receptors, suggesting that these long-term changes are dependent on receptor activation (Fig. 10B). Furthermore, long-term changes in receptor availability (B_max_) are eliminated by this mutation (Fig. 10D). However, changes in [^3^H]NMS affinity are still evident, which may be due to interference by persistently-bound xanomeline independent of receptor activation.

In conclusion, we have shown that acute as well as chronic xanomeline exposure results in long-term changes in M_1_ receptor binding and functional properties. Persistent binding of xanomeline elicits long-term changes in the receptor binding properties that are distinct from the profile obtained with carbachol, namely the appearance of a biphasic binding curve. We have demonstrated that pretreatment with high and low concentrations of xanomeline result in differential modes of receptor regulation. It is apparent that the effects observed at low concentrations of xanomeline are due, at least in part, to receptor down-regulation.

## Methods

### Materials

[^3^H]*N*-Methylscopolamine (82 Ci/mmol) was purchased from DuPont (Wilmington, DE); *myo*-[^3^H]inositol (85 Ci/mmol) was obtained from GE Healthcare (Little Chalfont, Buckinghamshire, UK); [^14^C]inositol-1-phosphate (300 mCi/mmol) was supplied by American Radiolabeled Chemicals (St. Louis, MO); Dulbecco's modified Eagle's medium was purchased from Invitrogen (Carlsbad, CA); geneticin was obtained from Calbiochem (San Diego, CA); and bovine calf serum was supplied by Hyclone (Logan, UT). Xanomeline tartrate was a generous gift from Eli Lilly & Co. (Indianapolis, IN); all other reagents were purchased from Sigma-Aldrich (St. Louis, MO).

### Cell Culture

Chinese hamster ovary (CHO) cells stably transfected with the human M_1_ muscarinic acetylcholine receptor (hM_1_) were provided by Dr. M. Brann, University of Vermont Medical School. The genes encoding the rat M_1_ wild-type (rM_1_) and a non-functional mutant (mutant^123^) muscarinic receptors were stably expressed in CHO cells [21]. All cells were grown at 37°C for 3–4 days in Dulbecco’s modified Eagle’s medium supplemented with 10% bovine calf serum and 50 µg/ml geneticin in a humidified atmosphere consisting of 5% CO_2_ and 95% air.

### Pretreatment regimen in whole cells

Cells were pretreated in monolayer at 37°C with culture medium in the absence or in the presence of xanomeline or carbachol (concentrations indicated in results) as follows: (1) control cells were incubated in the absence of agonist for 24 h; (2) cells were pretreated with agonist for 1 h; (3) cells were pretreated with agonist for 1 h and subsequently washed three times with iso-osmotic HEPES buffer (110 mM NaCl, 5.4 mM KCl, 1.8 mM CaCl_2_, 1 mM MgSO_4_, 25 mM glucose, 20 mM HEPES, 58 mM sucrose; pH 7.4; 340 mOsM) to remove unbound drug from the medium and allowed to incubate in culture medium in the absence of free agonist for 23 h; (4) cells were pretreated continuously with agonist for 24 h. After appropriate incubation periods, cells were washed with HEPES buffer three times before being used in binding or functional assays.

### Competition Binding Assays

CHO hM_1_, rM_1_, or mutant^123^ cells were seeded in 24-well plates and grown to 80–90% confluence prior to pretreatments. Control cells were incubated in monolayer in HEPES buffer with 0.2 nM [^3^H]*N*-methylscopolamine ([^3^H]NMS ) in the presence of increasing concentrations of xanomeline (1 nM to 100 µM) or carbachol (0.1 µM to 10 mM) for 1 h at 37°C. In order to assess the persistent effects of xanomeline or carbachol, cells were exposed to the previously described pretreatment regimen (concentrations indicated in results). Cells were subsequently incubated in monolayer with 0.2 nM [^3^H]NMS in HEPES buffer for 1 h at 37°C. Similar radioligand binding assays were performed in CHO hM_1_ cells following xanomeline pretreatments utilizing saturating concentrations of [^3^H]NMS (2.9 nM) or [^3^H]quinuclidinyl benzilate ([^3^H]QNB) (1.4 nM). In all cases, free radioligand was removed by surface washing and labeled cells detached by the addition of 1 M NaOH. The amount of radioactivity (disintegrations per minute) in each sample was determined by liquid scintillation spectrometry. Nonspecific binding was defined using 10 µM atropine.

### Saturation Binding Assays

CHO hM_1_, rM_1_, or mutant^123^ cells grown in tissue culture flasks were pretreated in monolayer at 37°C in the absence or in the presence of xanomeline (3 µM or 300 nM) as previously described. Cells were then harvested by trypsinization, followed by centrifugation (300×g, 3 min) and re-suspension of the pellet in HEPES buffer (three times). Subsequently, cells were incubated with increasing concentrations of [^3^H]NMS (0.02 to 4.5 nM) for 1 h at 37°C. Additional saturation experiments were conducted on CHO hM_1_ cells following 1-h pretreatment with atropine (10 µM) in the absence or in the presence of xanomeline (3 µM). Cells were washed extensively and allowed to incubate for 23 h in ligand-free media before being harvested. In a related set of experiments, atropine was added during the 23-h incubation following washing off free xanomeline. All experiments used 100,000 cells/assay tube in a total volume of 1 ml. Nonspecific binding was determined using 10 µM atropine. The reaction was terminated by filtration on Whatman GF/C filters (Whatman Schleicher and Schuell, Keene, NH) using a Brandel cell harvester (Brandel Inc., Gaithersburg, MD). Filters were washed three times with 4-ml aliquots of ice-cold saline and dried before radioactivity (disintegrations per minute) was measured using liquid scintillation spectrometry.

### Assay of phospho-inositide (PI) hydrolysis

CHO hM_1_, rM_1_, or mutant^123^ cells grown in tissue culture flasks were incubated in monolayer with culture medium containing *myo*-[^3^H]inositol (1 µCi/ml) for 24 h at 37°C. Labeled cells were harvested by trypsinization, centrifuged, and washed three times in HEPES buffer to remove unincorporated *myo*-[^3^H]inositol. Labeled cells were distributed to assay tubes (500,000 cells/tube), and allowed to incubate for 15 min at 37°C. Concentration-response curves for the stimulation of PI hydrolysis by xanomeline, carbachol or oxotremorine were constructed. Further experiments were designed to determine the effects of xanomeline pretreatment on agonist-stimulated PI hydrolysis. CHO hM_1_ cells were pretreated in monolayer with xanomeline (300 nM) or carbachol (10 µM), as previously described. Subsequently, concentration-response curves for the stimulation of PI hydrolysis by carbachol, oxotremorine, or xanomeline were constructed. Additional experiments were conducted following 1-h pretreatment with atropine (10 µM) in the absence or in the presence of xanomeline (3 µM). Cells were washed extensively and allowed to incubate for 23 h in ligand-free media before being harvested. In a related set of experiments, atropine was added during the 23-h incubation following washing off free xanomeline. Subsequently, concentration-response curves for the stimulation of PI hydrolysis by carbachol were constructed. In all cases, the reaction was allowed to proceed in the presence of 10 mM LiCl for 1 h at 37°C after the addition of agonist before being stopped with chloroform/methanol (2∶1) and centrifuged (450×g; 15 min).

Alternatively, cells were grown in 24-well plates and loaded with *myo*-[^3^H]inositol as described above. Following treatment with increasing concentrations of xanomeline as outlined previously, cells were washed three times in monolayer with iso-osmotic HEPES buffer. Subsequently, cells were exposed to 1 µM or 10 mM carbachol, 0.1 µM or 1 mM oxotremorine, or 0.03 µM or 0.1 mM xanomeline for 1 h at 37°C in HEPES buffer containing 10 mM LiCl. In order to determine the time course of xanomeline-induced persistent receptor activation and antagonism of carbachol-induced PI hydrolysis, cells were treated with 300 nM xanomeline for various times as indicated in results and washed. Cells were then incubated in the absence or in the presence of 10 µM carbachol for 1 h at 37°C in the presence of 10 mM LiCl. In all cases, the reaction was stopped with 0.3 M HClO_4_, neutralized with 0.15 M K_2_CO_3_, and samples were centrifuged (1500×g; 15 min).

For all experiments, [^14^C]inositol-1-phosphate was added to each sample as an internal recovery standard. Total inositol phosphates were separated by ion exchange chromatography (AG1-X8 resin). The amount of radioactivity (disintegrations per minute) in each sample was determined by liquid scintillation spectrometry and adjusted for ^14^C recovery.

### Dissociation Kinetics Assays

CHO hM_1_ cells grown in tissue culture flasks were pretreated in the absence or in the presence of xanomeline (10 µM) as previously described. Cells were harvested by trypsinization followed by centrifugation and resuspension in iso-osmotic HEPES buffer (three times). Cells were then incubated in HEPES buffer with a fixed concentration of [^3^H]NMS (0.5 nM) for 1 h at 37°C using 100,000 cells/assay tube. After this period, 10 µM atropine was added to inhibit reassociation of the radioligand. Nonspecific binding was measured in the presence of 10 µM atropine. The dissociation reaction was terminated by filtration as described above. The amount of bound radioactivity was measured at various time intervals to determine the dissociation rate of [^3^H]NMS.

### Data Analysis

Data were analyzed using Prism 4.0 (GraphPad Software Inc., San Diego, CA). Displacement binding isotherms were analyzed via nonlinear regression to derive estimates of IC_50_ (midpoint location or potency parameter). Data were fitted according to both one- and two-site mass-action binding models, and the better fit was determined by an extra sum-of-squares test. Due to the non-reversible nature of xanomeline binding, calculations of inhibition constants (K*_I_*) from IC_50_ values were not performed, as this conversion assumes reversible competitive interaction. Data from each complete saturation binding isotherm were analyzed after subtraction of nonspecific binding via nonlinear regression using Prism to derive individual estimates of B_max_ (total receptor density) and K*_D_* (radioligand-receptor equilibrium dissociation constant). Data from dissociation kinetic experiments were analyzed by Prism according to both monoexponential and biexponential dissociation models. Values of better fit based on an extra-sum-of-squares F-test were taken as estimates of *k*_off_ (radioligand dissociation rate constant).

In functional assays of PI hydrolysis, raw data were corrected for ^14^C recovery to account for individual column differences in efficiency. Individual concentration-response curve data were fitted to a four-parameter logistic function using Prism to obtain estimates of EC_50_ (half-effective concentration) and E_max_.

Data shown are the means ± standard error of the mean. Comparisons between mean values were made by unpaired *t-*tests or one-way ANOVA, as appropriate. A probability (*p*) value <0.05 was taken to indicate statistical significance.
